# Hydatid Cyst or Echinococcosis: A Comprehensive Review of Transmission, Clinical Manifestations, Diagnosis, and Multidisciplinary Treatment

**DOI:** 10.7759/cureus.63713

**Published:** 2024-07-02

**Authors:** Nitesh Badwaik, Pankaj Gharde, Raju K Shinde, Harshal Tayade, Pratik S Navandhar, Mihir Patil

**Affiliations:** 1 General Surgery, Jawaharlal Nehru Medical College, Datta Meghe Institute of Higher Education and Research, Wardha, IND

**Keywords:** imaging techniques, zoonotic disease, echinococcus granulosus, multidisciplinary treatment, diagnosis, clinical manifestations, transmission, parasitic infection, hydatid cyst, echinococcosis

## Abstract

Echinococcosis, a parasitic infection caused by Echinococcus tapeworms, can cause various symptoms depending on the location and size of the cysts. This article explores the complexities of echinococcosis, including its transmission cycle, clinical manifestations, diagnosis, and treatment approaches. The review highlights the challenges associated with diagnosing the different echinococcosis types, including cystic echinococcosis, alveolar echinococcosis, and polycystic echinococcosis. Each form of the disease necessitates a unique diagnostic approach that often combines serological tests, imaging techniques, and histological analysis. The article explores treatment options for each type of echinococcosis, including surgical resection, medication, and minimally invasive procedures such as puncture-aspiration-injection-reaspiration (PAIR). The article acknowledges current treatment methods' limitations and emphasises the need for further research into improved diagnostics, drug targets, and preventative measures. This review aims to provide a comprehensive overview of echinococcosis, encompassing its transmission, clinical presentation, diagnosis, and treatment modalities. By outlining the complexities of the disease and highlighting areas for future research, the article hopes to contribute to improved disease management and control. Key findings of the review include the identification of significant diagnostic challenges in differentiating between cystic, alveolar, and polycystic echinococcosis, the varying efficacy of treatment modalities such as surgical resection and PAIR, and the urgent need for further research into enhanced diagnostic methods, novel drug targets, and effective preventative strategies.

## Introduction and background

The parasitic ailment known as echinococcosis, also known as hydatid cyst, is brought on by Echinococcus tapeworms [1]. Alveolar echinococcosis and cystic echinococcosis are the two primary forms of the illness [1]. The less prevalent varieties are unicystic and polycystic echinococcosis [1]. Humans have known about echinococcosis for ages [1]. The global burden of echinococcosis is substantial, with over one million people affected at any given time [1,2]. The disease is prevalent in rural areas of developing countries where livestock farming is common and veterinary public health services are limited [2]. Endemic regions include parts of South America, the Mediterranean, Eastern Europe, the Middle East, Africa, and Central Asia [2]. Scholars from antiquity, including Hippocrates, Aretaeus, Galen, and Rhazes, acknowledged it as well [1,2]. Raw garlic and thymus vulgaris were among the herbs that were the basis of the suggested remedies [2]. The hydatid cysts associated with echinococcosis were shown to be of "animal" origin by Francesco Redi in the 17th century, marking the beginning of the investigation into the disease's aetiology [3]. Subsequently, Pierre Simon Pallas postulated in 1766 that the hydatid cysts discovered in sick humans were, in fact, tapeworm larvae [3,4]. It was determined that two calcified artefacts found in an adolescent's tomb near Amiens, Northern France, around the third or fourth century were most likely hydatid cysts [5,6]. Alveolar illness usually begins in the liver but can spread to other parts of the body, such as the lungs or brain [7]. The exact location and size of the cyst dictate which symptoms and signs manifest first. Typically, the illness starts with no symptoms and can last for years [7]. When the liver is impacted, the patient may have weight loss, stomach pain, and skin discolouration with a yellow tone due to jaundice [2]. Breathlessness, coughing, and chest pain are all possible side effects of lung illness [8]. An animal's infection can spread if it eats or drinks anything that has parasite eggs in it or comes into close contact with another affected species [1,4]. After eating an animal, such as a sheep or a mouse, that contains the cysts, commonly affected animals, such as dogs, foxes, and wolves, discharge the eggs in their faeces [9]. Treatment for potentially disease-carrying dogs and immunization of sheep are the two ways that cystic disease is prevented [1,2,4].

## Review

Search methodology

**Figure 1:**
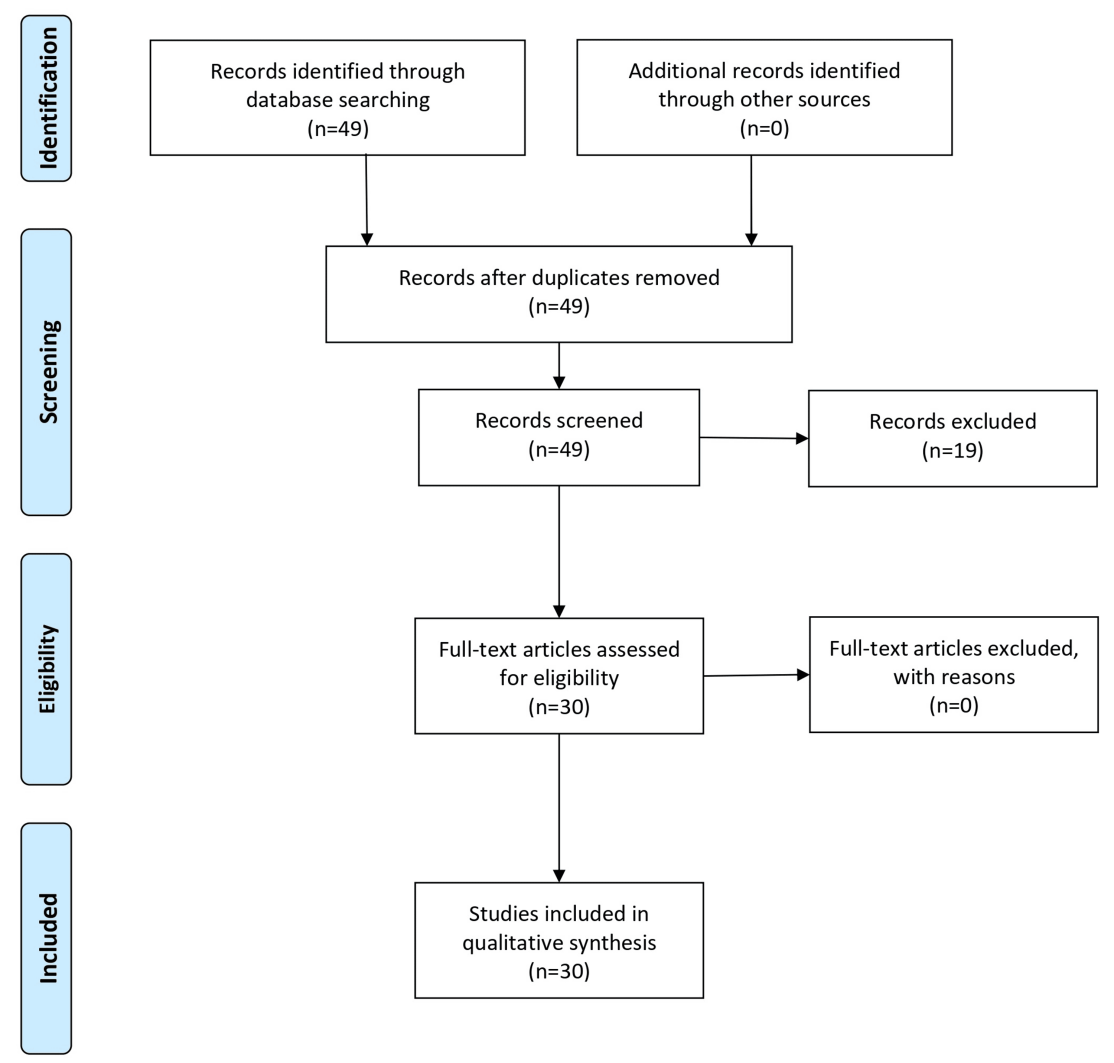
PRISMA flow chart PRISMA: Preferred Reporting Items for Systematic Reviews and Meta-Analyses The figure was created by Nitesh Badwaik.

The search methodology for the comprehensive review on echinococcosis encompassed a systematic approach following the Preferred Reporting Items for Systematic Reviews and Meta-Analyses (PRISMA) guidelines. The literature search involved electronic databases such as PubMed, Scopus, Web of Science, and Google Scholar. Keywords used in various combinations included "echinococcosis," "hydatid cyst," "Echinococcus," "clinical manifestations," "diagnosis," and "treatment." Relevant articles, reviews, clinical studies, and guidelines published in English were considered. Additionally, reference lists of selected articles were screened for additional relevant studies. The search strategy aims to comprehensively gather information on the transmission, clinical manifestations, diagnosis, and treatment approaches for different forms of echinococcosis. This systematic approach ensured the inclusion of diverse perspectives and up-to-date information to provide a thorough overview of the topic. The PRISMA flow chart is shown in Figure 1.

Clinical manifestations and symptoms of echinococcosis in humans

When the disease manifests in humans, Echinococcus granulosus, E. multilocularis, E. oligarthrus, and E. vogeli are found in the liver (75% of cases), the lungs (5-15% of cases), and other organs such as the spleen, brain, heart, and kidneys (10-20% of cases) [1,10]. When a person has cystic echinococcosis due to an E. granulosus infection, the illness manifests as a slowly expanding tumour within the body [11]. Alveolar and polycystic echinococcosis patients also have these slowly expanding lumps, which are frequently referred to as cysts [11,12]. Cystic echinococcosis patients typically have spherical, single-chambered cysts that are only present in one place of the body [11,12]. The clear substance inside these cysts is called hydatid fluid [4]. Although the cysts in patients with polycystic and alveolar echinococcosis and cystic echinococcosis are similar, the former group typically has many compartments and exhibits infiltrative rather than expansive growth [1,13].

Symptomatic Variability and Complications Based on Cyst Location

Depending on where the cyst is located in the body, the patient may experience no symptoms, even if the cysts are minuscule [12,14]. If the patient has symptoms, a lot will rely on the location of the cysts [4]. For example, if the patient has lung cysts and is experiencing symptoms, they may cough, feel short of breath, or have chest pain [8,13]. However, if the patient has liver cysts and is symptomatic, they may also have jaundice, fever, atypical abdominal discomfort, hepatomegaly with an abdominal mass, and stomach pain [9]. Additionally, if the cysts burst while it was still inside the body, either because of bodily trauma or a surgical procedure to remove them, they would probably result in fever, pruritus, swelling of the lips and eyelids, dyspnea, stridor, rhinorrhea, and anaphylactic shock [1,12,15].

In contrast to intermediate hosts, definitive hosts typically experience minimal damage from the infection [2]. Occasionally, the parasite's extreme demand can result in the host lacking specific vitamins and minerals [16]. All Echinococcus species have an incubation period that lasts from months to years or even decades [16]. It mainly relies on where the cyst is located in the body and how quickly it is expanding [4,16].

The complex life cycle and transmission pathways of echinococcus

The worm's life cycle necessitates definitive and intermediate hosts, complicating Echinococcus infections [10]. Herbivores such as sheep and cattle are typically intermediate hosts, whereas definitive hosts are typically carnivores such as dogs [17]. Because they are typically a dead end for the parasite infection cycle - unless wolves or dogs consume them after death - humans serve as accidental hosts [7,10,12]. A permanent host's small intestine is home to an adult worm. The final host excretes the eggs released by a solitary gravid proglottid [18]. After that, an intermediary host consumes the egg. Following hatching in the small intestine of the intermediate host, the egg releases an oncosphere that passes past the intestinal wall and into the circulatory system, reaching the liver and lungs, among other organs [5,17,19]. When the oncosphere penetrates these organs, it turns into a cyst. The cyst then gradually grows, forming daughter cysts inside it and protoscolices, or juvenile scolices [1]. Following ingestion of the cyst-containing organs of the infected intermediate host, the infection also infects the definitive host [1]. The protoscolices adhere to the colon following intake [20]. After that, they mature into adult worms, and the cycle repeats.

All pathogenic Echinococcus species are transmitted to intermediate hosts by eating their eggs and to definitive hosts by consuming contaminated organs that contain cysts [19]. By coming into contact with dirt, mud, or hair from animals that carry eggs, humans unintentionally become intermediate hosts for the infection [1,5,18]. There are no biological or mechanical vectors for any Echinococcus species' adult or larval stages. However, coprophagic flies, carrion birds, and arthropods can function as mechanical vectors for the eggs [17,18,21]. Echinococcus life cycle and transmission is depicted in Table 1.

**Table 1:** Echinococcus life cycle and transmission Stage: Lifecycle stage of the parasite. Host: Type of host the parasite infects (definitive or intermediate). Description: Brief explanation of the parasite's behavior or location at this stage. Transmission: How the parasite moves between hosts (environment, accidental, or mechanical vectors). Environment: Transmission through contaminated soil/dirt. Accidental: Transmission to humans through accidental contact. Mechanical vectors: Transmission by organisms carrying eggs without being infected themselves. (Adult/larval): Indicates the vector does not harbor the parasite itself (adult or larval stage). (Eggs): Indicates the vector carries the parasite eggs on its body. References: [7,10,12,17,19,21]. The table was created by Nitesh Badwaik.

Stage	Host	Description
Adult worm	Definitive (carnivore)	Lives in small intestine, releases eggs
Eggs	Environment	Excreted by definitive host, contaminate soil/dirt
Egg (hatching)	Intermediate (herbivore)	Ingested by intermediate host, hatches in small intestine
Oncosphere	Intermediate	Travels through bloodstream, reaches organs (liver, lungs)
Cyst	Intermediate	Develops in organs, grows, forms daughter cysts and protoscolices
Protoscolices (infection)	Definitive	Ingested by definitive host through infected organs
Adult worm	Definitive	Develops in colon, restarts cycle
Transmission (intermediate)	Environment	Eggs contaminate soil/dirt, ingested by intermediate host
Transmission (accidental)	Human	Contact with eggs on soil/dirt or animal hair
Mechanical vectors	None (adult/larval)	
Mechanical vectors (eggs)	Flies, birds, arthropods	Passively carry eggs on bodies

Comprehensive diagnostic approaches for echinococcosis

Diagnostic Methods for Cystic Echinococcosis

Any kind of echinococcosis requires a combination of methods for definitive diagnosis, including serology, histology, nucleic acid detection, and imaging techniques [22]. Imaging is the primary approach used to diagnose cystic echinococcosis; serology tests, such as latex agglutination, immunoblots, ELISA (enzyme-linked immunosorbent assay), indirect hemagglutination, or immunoblots, using antigens specific to E. granulosus, validate the imaging results [23,24]. When it comes to cystic echinococcosis, ultrasonography is the preferred imaging modality because, in addition to seeing the cysts in the body's organs, it is affordable, non-invasive and provides immediate findings [23]. MRIs and CT scans can be utilized in addition to ultrasonography. At the same time, an MRI is usually preferred over a CT scan for diagnosing cystic echinococcosis because it provides a clearer view of the liquid areas inside the tissue [11,25].

Diagnostic Methods for Alveolar Echinococcosis

Similar to cystic echinococcosis, the preferred imaging method for alveolar echinococcosis is ultrasonography, which is typically supplemented by CT scans because the latter can identify the greatest number of lesions and calcifications that are indicative of alveolar echinococcosis [12]. Although CT scans are preferred, MRIs can also be performed in conjunction with ultrasonography. Alveolar echinococcosis is primarily diagnosed by imaging, much as cystic echinococcosis [26]. The imaging results are confirmed by the same kinds of serologic testing, which are now specific for E. multilocularis antigens [26]. It is also noteworthy that alveolar echinococcosis is more accurately diagnosed with serologic testing than cystic echinococcosis since there are more antigens unique to E. multilocularis for alveolar echinococcosis, which makes the test more reliable [24]. Alveolar echinococcosis can also be diagnosed by serology, imaging, or polymerase chain reaction (PCR)-confirmed E. multilocularis infection or histological examination of a tissue sample from the patient [10].

Diagnostic Methods for Polycystic Echinococcosis

As with the diagnosis of cystic and alveolar echinococcosis, polycystic echinococcosis is determined by imaging methods, including CT and ultrasonography scans, which identify polycystic formations in the body [4]. Imaging is not the recommended method of diagnosis, though, as the currently accepted approach involves isolating protoscoleces during surgery or after a patient passes away and then identifying the conclusive characteristics of E. oligarthrus and E. vogeli in these isolated protoscoleces [27]. This is the primary method used to diagnose PE, yet recent research indicates that PCR may be able to detect E. oligarthrus and E. vogeli in human tissues [5,8]. The limited availability of genetic sequences specific to E. oligarthrus or E. vogeli makes PCR the sole practical method for diagnosing polycystic echinococcosis [27]. Diagnostic approaches for echinococcosis are described in Table 2.

**Table 2:** Diagnostic approaches for echinococcosis Echinococcosis type: This refers to the specific kind of echinococcosis infection a patient has. There are four main types: cystic, alveolar, polycystic, and unicystic. Preferred imaging: This indicates the initial imaging modality that doctors typically recommend for diagnosing a specific echinococcosis type. Additional imaging modality: This lists any other imaging techniques that might be helpful, in addition to the preferred modality, for diagnosing the echinococcosis type. Serologic test antigen: This specifies the particular antigen used in a blood test to identify the type of echinococcosis infection. Not all types require a serologic test. Definitive diagnosis: This explains how doctors definitively diagnose each echinococcosis type. It usually involves a combination of imaging tests and serologic tests, but in some cases, isolating protoscoleces, the larval stage of the parasite, might be necessary. CT scan: Computed Tomography Scan; MRI: Magnetic Resonance Imaging; NA: Not Applicable Table References: [10,11,12,22-26] The table was created by Nitesh Badwaik.

Echinococcosis Type	Preferred Imaging	Additional Imaging Modality	Serologic Test Antigen	Definitive Diagnosis
Cystic	Ultrasonography	MRI, CT Scan	E. granulosus	Imaging + Serologic Test
Alveolar	Ultrasonography	CT Scan	E. multilocularis	Imaging + Serologic Test
Polycystic	Ultrasonography, CT Scan	NA	NA	Isolating Protoscoleces (E. oligarthrus/E. vogeli)

Treatment and management of echinococcosis

Treatment Options for Cystic Echinococcosis

Currently, there are numerous options for treating echinococcosis. Smaller, simpler cysts (less than 5 cm) respond well to albendazole treatment, whether or not praziquantel is added [28]. Merely 30% of cysts disappear when treated medically. Two times a day is the recommended dosage for albendazole for one to four months [9,27,28]. Mebendazole is an alternative to albendazole, which must be taken for a minimum of three to six months [1]. Larger liver cysts (> 10 cm), cysts that could rupture, and/or complex cysts should all be surgically treated [4,25]. Using a laparoscopic procedure results in a high rate of recovery with low morbidity and death [29]. Since total cystopericystectomy has a lower incidence of biliary fistula, postoperative abdominal infection, and overall morbidity, it is the preferred radical approach [25]. In endemic regions where nonspecialist surgeons do surgery, conservative procedures are recommended.

As an alternative to surgery, puncture-aspiration-injection-reaspiration (PAIR) [19] is a novel approach [30]. Three phases make up the minimally invasive PAIR procedure, which includes puncture and needle aspiration of the cyst, injection of a scolicidal solution for 20-30 minutes, and finally cyst reaspiration and final irrigation [9,30]. Albendazole or mebendazole is usually taken by PAIR patients starting seven days prior to the surgery and continuing for 28 days following it [30]. It is recommended for patients who are not responding to medical treatment, for recurrence following surgery, for inoperable cases, and for people who refuse surgery [27]. Numerous studies indicate that PAIR along with medicinal therapy is superior to surgery in terms of morbidity and mortality as well as disease recurrence [9,30].

Studies and research are presently being conducted on a novel form of treatment called percutaneous thermal ablation (PTA), which uses a radiofrequency ablation (RFA) device to destroy the germinal layer of the cyst [1,22]. Since this medication is still relatively new, considerably more testing is necessary before it is applied broadly.

Treatment Options for Alveolar Echinococcosis

The only certain treatment for alveolar echinococcosis is surgical cyst ectomy followed by chemotherapy (with albendazole and/or mebendazole) for up to two years following surgery [9,16]. Chemotherapy alone, however, can also be utilized in cases that are incurable. Albendazole in two doses or mebendazole in three doses could be used in a chemotherapy-only regimen [28]. People are frequently put on chemotherapy for longer periods of time because it is not always guaranteed to eradicate the disease completely (i.e. more than six months, years) [28]. Liver transplants are being investigated as a therapeutic option for alveolar echinococcosis in addition to surgery and chemotherapy; however, this procedure is extremely risky because it frequently results in echinococcosis re-infection in the patient [9,13].

Treatment Options for Polycystic Echinococcosis

Treatment for polycystic echinococcosis is less established than for cystic and alveolar echinococcosis because the condition is limited to a certain region of the world, is poorly characterized, and affects fewer individuals [27]. For the first two forms of echinococcosis, surgical cyst ectomy was the preferred course of treatment; however, for polycystic echinococcosis, chemotherapy is the suggested course of action [5,27]. Albendazole is the recommended medication; however, if the course of treatment is going to be prolonged, mebendazole may also be utilized [27]. Surgery is only recommended if chemotherapy is unsuccessful or if the lesions are very tiny. Treatment and management of echinococcosis are described in Table 3.

**Table 3:** Treatment and management of echinococcosis Type of echinococcosis: This refers to the specific kind of echinococcosis infection, which can be cystic (further classified by size and complexity) or alveolar, and a less common type, polycystic. Treatment options: This outlines the different medical approaches used to treat each type of echinococcosis. Description: This provides a brief explanation of each treatment option, including its effectiveness and potential downsides. PAIR: Puncture-Aspiration-Injection-Reaspiration; PTA: Percutaneous Thermal Ablation Table References: [4,9,13,16,25,27,28,30] The table was created by Nitesh Badwaik.

Type of Echinococcosis	Treatment Options	Description
Cystic (smaller, simpler cysts)	Albendazole therapy (with or without praziquantel)	Medication for 1-4 months; effective for cysts < 5 cm; 30% cure rate
Cystic (larger, complex cysts)	Surgery (laparoscopic or open)	Total cystopericystectomy preferred; high cure rate, low complication rate
Cystic (alternative to surgery)	PAIR (puncture-aspiration-injection-reaspiration)	Minimally invasive procedure; combined with albendazole/mebendazole medication
Cystic (under research)	Percutaneous thermal ablation (PTA)	Uses radiofrequency to destroy cyst; new treatment, more research needed
Alveolar	Surgery (cyst ectomy) + chemotherapy (albendazole/mebendazole)	Surgery followed by up to 2 years medication
Alveolar (inoperable cases)	Chemotherapy only (albendazole or mebendazole)	Long-term treatment (over 6 months)
Alveolar (under research)	Liver transplant	High-risk procedure, frequent re-infection
Polycystic	Chemotherapy (albendazole or mebendazole)	Albendazole preferred; surgery only if ineffective

Echinococcosis treatment with modern surgical and imaging innovations

In recent years, surgical treatments for the removal of echinococcosis caused by E. granulosus (cystic echinococcosis) and E. multilocularis (alveolar echinococcosis) have advanced significantly [1]. Laparoscopy and thoracoscopy are two examples of minimally invasive techniques that have transformed surgical treatment by giving benefits such as less postoperative pain, shorter hospital stays, and faster recovery times [29]. Robotic-assisted surgery is a new discipline that enhances control, dexterity, and precision while removing cysts from difficult-to-reach anatomical areas [15,22,30]. Modern imaging modalities, such as MRI, CT-guided navigation, and intraoperative ultrasound, improve cyst localization and removal accuracy, reduce complications, and ensure complete cyst excision [11,25,26]. Endoscopic techniques, such as percutaneous endoscopic cyst draining, provide a less invasive alternative to open surgery [30].

Hybrid surgical methods, which combine open, laparoscopic, and endoscopic procedures, improve each patient's recovery time and treatment efficacy. With advances in sclerotherapy agents, the PAIR method offers a less invasive treatment option for liver cysts while improving success rates and safety profiles [9,19]. Other methods of removing echinococcal cysts include cryosurgery and RFA, particularly useful when the cysts are inoperable or reoccurring [30]. In addition, individualized models of a patient's anatomy and cysts may be made utilizing 3D-printing technology for pre-surgical planning [11,12,25]. This allows surgeons to plan their strategies ahead of time, potentially saving time and improving outcomes. These advances in surgical procedures and technology not only increase the precision and efficacy of cyst removal, but they also improve patient outcomes, reduce morbidity, and shorten recovery time, indicating a significant improvement in the therapy of this challenging parasitic condition [29].

## Conclusions

Echinococcosis, a parasitic disease caused by Echinococcus tapeworms, can manifest with diverse symptoms depending on the cyst's location and size. Common symptoms include weight loss, abdominal pain, jaundice, cough, and shortness of breath. Diagnosis involves a combination of imaging techniques and blood tests specific to the Echinococcus species. Treatment options such as medications, surgery, or even liver transplants for severe cases, depend on the type and severity of infection. While advancements have been made, echinococcosis remains a public health concern. Future research directions include developing more accurate diagnostic tests, exploring new drug targets, minimally invasive surgical techniques, and effective preventive measures to break the transmission cycle. These advancements can lead to improved diagnosis, treatment, and ultimately better disease control.
